# The Efficacy and Therapeutic Alliance of Augmented Reality Exposure Therapy in Treating Adults With Phobic Disorders: Systematic Review

**DOI:** 10.2196/51318

**Published:** 2023-11-30

**Authors:** Safa Hasan, Hamid Alhaj, Athanasios Hassoulas

**Affiliations:** 1 Cardiff University Cardiff United Kingdom; 2 University of Sharjah Sharjah United Arab Emirates

**Keywords:** augmented reality, virtual reality, anxiety disorders, phobic disorders, exposure therapy, augmented reality exposure, phobia, excessive fear, prevalence, technology, cost-effectiveness, fear, phobia, phobic

## Abstract

**Background:**

Phobic disorders are characterized by excessive fear of a stimulus that can affect the quality of a patient’s life. The lifetime prevalence in adults is 7.7% to 12.5%. The current literature provides evidence-based inferences about the effectiveness of in-vivo exposure therapy (IVET) in treating phobia. However, this method can put the therapist and the client in danger, with high drop out and refusal rates. A newer approach for exposure therapy using augmented reality technology is under assessment.

**Objective:**

This systematic review investigated the novel technology’s efficacy, cost-efficacy, and therapeutic alliance in treating adults with phobia.

**Methods:**

An extensive search was conducted using 4 major databases (MEDLINE, PsycINFO, Embase, and Scopus) using a comprehensive list of synonyms for augmented reality exposure therapy (ARET) and phobic disorders. The search targeted any randomized control trial testing ARET in adults with phobic disorders up to August 8, 2022.

**Results:**

A total of 6 studies were included, with 208 participants providing results. Studies investigating the efficacy of ARET compared to no intervention showed significant results (*P*<.05) in the ARET group improvement. Head-to-head comparative studies comparing ARET to IVET showed no significant difference (*P*>.05) in the effectiveness and therapeutic alliance between both therapies. Further, the results demonstrated that the ARET group had a better long-term effect than IVET, with the ability to put the patients in more situations to face the feared object.

**Conclusions:**

The current data suggest clinically significant efficacy and a promising therapeutic alliance of ARET. However, no data are available investigating the cost-effectiveness of ARET. Further research is warranted to ascertain ARET’s cost-effectiveness and examine its efficacy in other populations and anxiety conditions.

## Introduction

### Background

Phobic disorders are classified under the umbrella of anxiety and fear-related disorders and are characterized by excessive and abnormal fear or anxiety toward specific things or situations [1]. Fear is a psychological response to current stimuli, while anxiety is more concerned with future perceived anticipated stimuli or threats [1,2]. In the United States, adults’ lifetime prevalence of specific phobias is estimated to range between 7.7% and 12.5% [3].

Notably, the first line of management in phobic disorders is psychotherapy; however, it can be augmented with medications [4,5]. Different modalities of cognitive behavioral therapy (CBT) can be used, although CBT with exposure is believed to be the most effective treatment [6,7]. CBT targets the patients’ way of cognition and thinking to help them change their behaviors toward a specific issue and help them develop new techniques (deconditioning and counterconditioning) for dealing with their problems [7,8]. To avoid the limitations of in-vivo exposure therapy (IVET), like high dropout rate and low treatment acceptance [6], newer methods are being developed, including the virtual reality exposure therapy (VRET) [9]. Virtual reality (VR) technology was noted to assist in the exposure therapy with the aim of encountering the object or the case in a safer environment, and it will help create exposures that are hard to test in real life [9,10].

VR technology has been extensively studied, with evidence suggesting the superiority of VR over traditional psychotherapies in multiple areas of psychiatric disorders [11]. Riva et al [12] discussed the different uses of VR in psychiatric illnesses, and based on 27 systematic reviews and meta-analysis, they concluded the effectiveness of VR in the treatment of anxiety disorders, posttraumatic stress disorder, eating disorders, obesity, and pain management.

In the past decade, augmented reality (AR) has been routinely used in various fields such as medicine, entertainment, maintenance, design and architecture, teaching, and cognitive and motor rehabilitation [13]. AR has been shown to be effective in some regions of medicine, including improving physical activity and learning in autism [14,15]. Given that AR enhances the real-world environment with computer-generated sensory input, such as images, sounds, or other information, it has been proposed that they may be particularly useful in the treatment of phobic disorders.

Recent studies comparing the 2 technologies appear to support the advantage of AR compared with VR in particular psychiatric conditions [16]. While both VR and AR have shown promising results in treating anxiety disorders, it is thought that AR may have the advantage of providing a less intense form of exposure therapy, since the patient is still in the real world but with the added support of computer-generated elements. Vinci et al [17] discussed AR as an effective novel technology for treating substance dependence and anxiety disorders. In addition, Riva et al [12] and Vinci et al [17] suggested that AR adds to the benefits of normal clinical psychology and discussed the potential effectiveness of AR. However, limited data were available to provide any conclusive results for the efficacy of AR in various types of psychiatric disorders.

The therapeutic alliance is very crucial in the management of psychiatric disorders and is considered a key factor in the success of therapy. A strong therapeutic alliance is characterized by mutual trust, respect, and collaboration between the therapist and client, with the goal of achieving the client’s therapeutic goals. Therapeutic alliance can be tested by 3 main domains of a good alliance [18,19]. In addition, Tracey and Kokotovic [18] described the 3 domains: goal, task, and bond. The therapeutic alliance is one of the essentials for a good outcome in psychotherapy; a good quality therapeutic alliance is linked to the higher success of the psychotherapeutic approach [19]. So, to agree on the efficacy of any intervention, a good-quality therapeutic alliance should occur. Based on the current literature, the therapeutic alliance in technology-mediated psychotherapy is still questioned [20].

### Objectives

The main objective of this systematic review is to understand how effective augmented reality exposure therapy (ARET) is in treating phobic disorders. In addition, we aimed to investigate the best strategy to be used in exposure therapy with a lower recurrence rate, lower cost, and higher acceptance by the patients. Thereby, therapeutic alliance, cost-effectiveness, and efficacy of AR will be investigated.

## Methods

### Search Strategy

A comprehensive search included 4 databases. MEDLINE was our primary database to test for the eligibility of the search strategy, and the search strategy was duplicated on Embase, PsycINFO, and Scopus. The keywords included Phobia OR Phobia* OR “Phobic AND disorders” OR Agoraphobia AND “augmented AND reality” OR “augmented AND reality AND exposure AND therapy” OR “ARET” OR “mixed AND reality”. Furthermore, gray literature was not included in the search to guarantee better quality papers as the grey literature is not peer-reviewed literature. However, excluding the gray literature can decrease the new studies done in this area and increase the risk of publication bias in this study [21].

### Definitions

AR is defined as a technology that superimposes an overlay of simulated objects [22,23]. AR psychotherapy was defined as any psychotherapy that uses AR technology, like ARET. Phobic disorders were defined according to standardized diagnostic criteria, namely Diagnostic and Statistical Manual of Mental Disorders (DSM) or International Classification of Diseases (ICD).

### Inclusion and Exclusion Criteria

The selection of the studies followed the Population, Intervention, Comparison and Outcomes (PICOS) approach to focus on a specific question [24]. (1) Inclusion criteria: Participants are adolescents and adults between 15 and 75 years of age, those who met the criteria for the diagnosis of specific phobia and are eligible for treatment, and those with no other comorbid disorders. The intervention included any psychotherapy that uses AR technology, like ARET. The comparison groups include groups that use VR technology for exposure therapy, groups treated with the usual real-life exposure therapy (treatment as usual [TAU]), groups treated using CBT or any psychotherapy, or waiting lists. The outcome measures are multiple, including qualitative questionnaires (testing the behaviors of avoidance and the therapeutic alliance) and quantitative measures measuring the physiological effect of fear and anxiety, such as heart rate, electrocardiograms (ECGs), and skin conductance recordings (SCR). Finally, randomized control trials are the design targeted. (2) Exclusion criteria: uncompleted studies or studies that did not have a comparison group.

### Procedure

**Figure 1:**
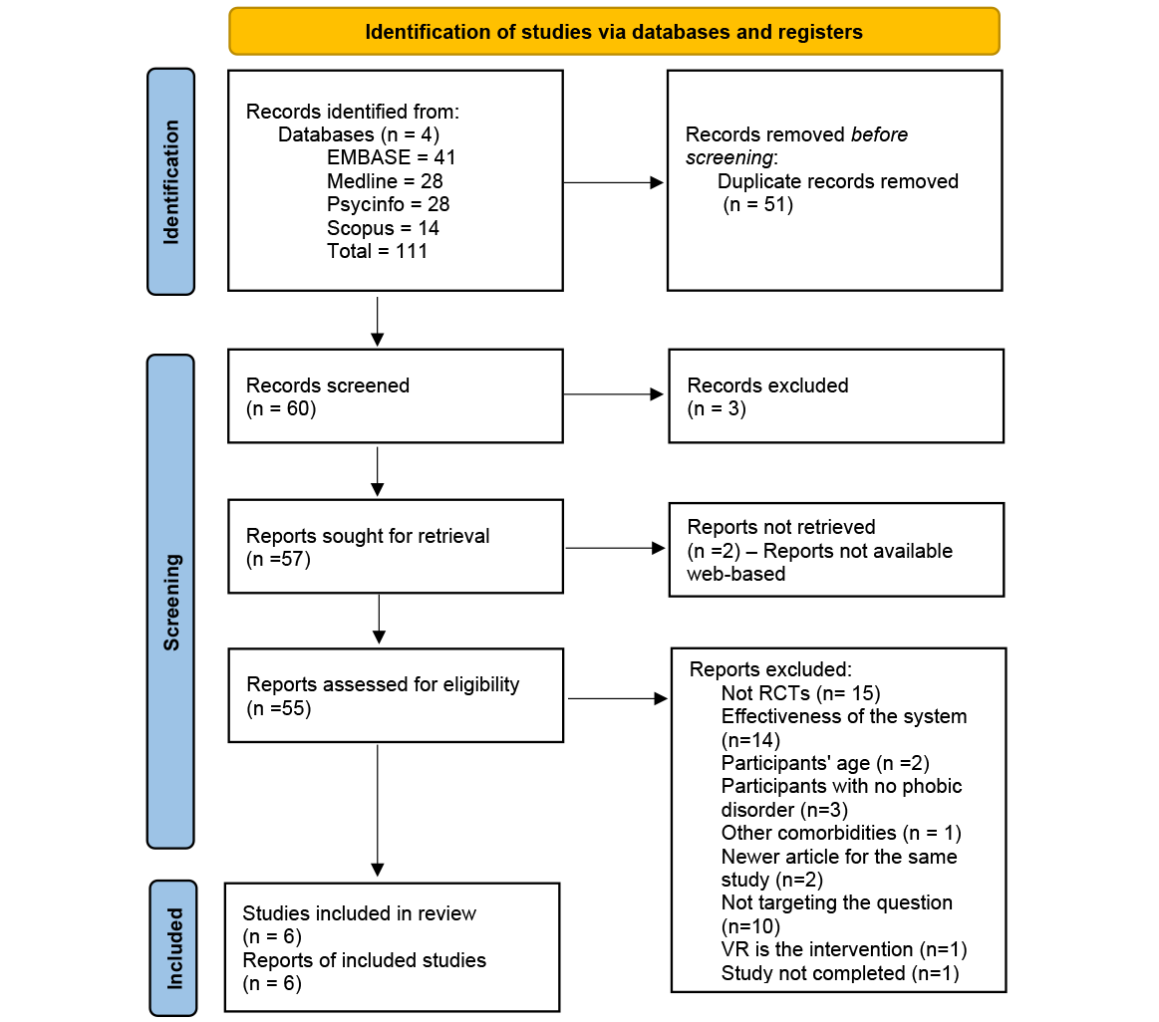
PRISMA diagram illustrating the selection procedure. PRISMA: Preferred Reporting Items for Systematic Reviews and Meta-Analyses; RCT: randomized controlled trial; VR: virtual reality.

Initially, the MEDLINE database was searched for using AR technology to treat phobic disorders; a preliminary result of 122 records was retrieved and screened for relevancy. Some synonyms were removed from the search strategy as the results revealed unrelated studies. Later, a second run was done and included relevant studies. The search keywords were duplicated in all the databases. All the records were imported into EndNote 20 (Clarivate Analytics) and were screened for duplication and eligibility for inclusion (as per the criteria above). The abstracts were used for a primary screening; however, if the decision was unclear based on the abstract, a full study was retrieved to determine the eligibility. Later, after the final screening, the included papers’ information was extracted into Excel (Microsoft Corp) sheets and Microsoft tables to help in the comparison; the quality assessment tools used helped select the needed data to extract and also, to map out the number of records and reports screened, retrieved, excluded, and included in this study [25]. The PRISMA (Preferred Reporting Items for Systematic Reviews and Meta-Analyses) flow diagram was filled during each step of this study (Figure 1). Furthermore, the review included a qualitative synthesis of the available studies in this research field. The qualitative synthesis included a very detailed assessment of each study’s findings. And multiple bias assessments were used to assess for common types of bias, including the Critical Appraisal Skills Programme (CASP) checklist, the Jadad scale, and the bias assessment table by Boland et al [24]. While a quantitative synthesis was supposed to be carried out, it was not done due to the unavailability of the raw data.

## Results

### Overview

A total of 111 studies were found. After the first screening, 60 records remained and were screened, with 3 records being irrelevant, and 2 records being unavailable on the internet; 55 studies were screened for eligibility. A total of 7 studies met the inclusion criteria; however, 1 study met the exclusion criteria and was removed. A total of 208 participants were studied in 6 studies, excluding Toffolo et al [26], with 112 participants, as the study is not yet completed. Furthermore, the control group of the 7 studies was the following: 4 studies (4/6) had IVET as the control group, and 2 studies (2/6) had the waiting list as a control group.

Furthermore, different outcome measures were used to conclude the results, as displayed in Table 1. These include the Behavioral Avoidance Test (BAT), Fear of Spiders Questionnaire (FSQ), Spider Phobia Beliefs Questionnaire (SPBQ), and Working Alliance Inventory-Short (WAI-S). These are the most used validated qualitative questionnaires in studies with spider phobia. BAT evaluates the ability of the participants to change their maladaptive behavior (avoidance). At the same time, FSQ and the SPBQ are ideally used together to cover the gaps in SPBQ [27]. Finally, WAI-S is the short version of the Working Alliance Inventory, which is a validated scale to understand the therapeutic alliance between the therapist and the patients. Moreover, some studies used quantitative outcome measures like SCR; SCR measures the autonomic arousal caused by fear and anxiety. All studies have significant results showing ARET’s good efficacy (and continuous efficacy) in treating phobic disorders. In addition, studies concerned about the therapeutic alliance of AR showed a good alliance between therapists and participants with no significant overall differences between ARET’s and IVET’s therapeutic alliance.

Studies comparing AR to the IVET (4/6) showed nonsignificant results between the 2 modalities in both the efficacy and the therapeutic alliance (*P*>.05), suggesting good effectiveness, feasibility, and a therapeutic alliance of AR. And the studies comparing the novel technology with a waiting list (2/6) showed significant statistical results with a *P*<.05, suggesting a good efficacy of ARET compared to nothing being done. The below Table 1 illustrates summary of the studies: clinical condition, participants, interventions, measures, and outcomes.

**Table 1:** Summary of the key studies included in the review with details of interventions, measures, and outcomes.

		Clinical condition	Participants	Intervention	Measures	Outcomes
**IVET^a^ for control**						
	Botella et al [28], Spain	Specific phobia (cockroaches phobia and arachnophobia)	In vivo exposure (N=31) augmented reality system (N=32)	One intensive session (up to 3 h long)—clinical settings	BAT^b^, FSQ^c^, SPBQ^d^, Fear-MTB^e^, and CSS^f^	Directly posttreatment measures showed IVET superiority in some measures. However, follow-up at 3 and 6 months showed nonsignificant results between IVET and ARET^g^. ARET is effective in treating small animal phobia. ARET is accepted and well-tolerated by patients.
	Wrzesien et al [29], Spain	Specific phobia (cockroaches phobia and arachnophobia)	N=22 randomly assigned to IVET or ARET	One intensive session (up to 3 h long)—clinical settings	BAT and WAI-S^h^	Nonsignificant differences between ARET and IVET in the overall therapeutic alliance.
	Wrzesien et al [30], Spain	Specific phobia (cockroaches Phobia and arachnophobia)	N=20, randomly assigned to IVET or ARET	One intensive session (up to 3 h long)—clinical settings	TCS^i^, WAI-S, and BAT	Nonsignificant differences between ARET and IVET in the overall therapeutic alliance. TCS was correlated to WAI-S and BAT scores. TCS helped in understanding the significant differences of WAI-S subscales with an overall limited effect on the alliance. TCS can be used in future research as a scale to test for therapeutic alliance in technology mediated therapy.
	Wrzesien et al [31], Spain	Specific phobia (cockroaches phobia and arachnophobia)	N=12, randomly assigned to IVET or ARET	One intensive session (up to 3 h long)—clinical settings	Participants’ outcome measures: BAT, WAI-S, and scales for anxiety, avoidance, and beliefs. The therapists' measures: capacity of the ARET. Usefulness and the frequency of use. Scale for therapeutic alliance. Sessions recorded.	ARET and IVET showed similar effectiveness on a clinical basis. Both groups were able to interact with a live feared object after the session.
**Waiting list for control**						
	Javanbakht et al [32], USA	Specific phobia (arachnophobia)	ARET (n=13) waiting list control (n=12)	The sessions conducted in the clinics aimed to gradually increase the intensity until the patient SUDS^j^ score was below 4. This was followed with nonclinical settings for 1 month on patients’ own base of exposure using AR^k^ app.	FSQ, SPBQ, CEQ^l^, BAT, SUDS, biosignal measures: SCR^m^	ARET group were able to touch a live tarantula or the tank after 1 session, while waiting list group stayed a minimum of 1 meter away from the tank. ARET efficacy on decreasing the symptoms remained the same or improved at 1 month follow.
	Zimmer et al [33], Switzerland	Specific phobia (arachnophobia)	Gamified AR spider exposure app (n=33), control group: waiting list (n=33)	Patients were trained in a clinical setting using 1 session of gradual exposure, then they were sent home with the app access to do 6 sessions of 30 minutes each, within 2 weeks period.	SUDS, FSQ, BAT, SBQ^n^, GSE^o^, 1 question to assess self-reported fear reduction of spiders.	The repeated use of nonclinical settings reduced subjective fear in all measures and was effective at a low cost. Nonclinical settings exposure was short and time-saving. Exposure to a simulated object was safe in a nonclinical setting.

^a^IVET: in-vivo exposure therapy.

^b^BAT: Behavioral Avoidance Test.

^c^FSQ: Fear of Spiders Questionnaire.

^d^SPBQ: Spider Phobia Beliefs Questionnaire.

^e^Fear-MTB: Main Target Behavior (Fear).

^f^CSS: Clinician Severity Scale.

^g^ARET: augmented reality exposure therapy.

^h^WAI-S: Working Alliance Inventory-Short.

^i^TCS: Therapeutic Collaboration Scale.

^j^SUDS: Subjective Units of Distress Scale.

^k^AR: augmented reality.

^l^CEQ: Credibility/Expectancy Questionnaire.

^m^SCR: skin conductance recordings.

^n^SBQ: Spider phobia beliefs questionnaire.

^o^GSE: General Self-Efficacy Scale.

### Bias Assessment of the Included Studies

Botella et al [28] and Wrzesien et al [30] studies fulfilled most of the requirements for high-quality research in the CASP checklist and rated 4 out of 5 on the Jadad scale. However, Wrzesien et al [29] and Wrzesien et al [31] did not report most of the sections in the CASP checklist and rated 1 out of 5 and 2 out of 5 on the Jadad scale, respectively; a lower quality and higher level of bias results are expected. Javanbakht et al [32] and Zimmer et al [33] scored a 5 out of 5 on the Jadad scale and filled out the CASP checklist perfectly; this means both are high-quality studies that can be reliable in their results with a lower chance of bias.

## Discussion

### AR Efficacy Compared With Other Modalities

Based on the results of this review, despite knowing that the spiders are simulated and unreal, ARET showed a statistically significant outcome in treating small animal phobia. Participants being treated by ARET in all 6 completed studies showed a substantial improvement in their phobic symptoms; each study used different outcome measures, but all the measurements were validated in testing the phobic symptoms. ARET showed no statistical differences (*P*>.05) compared to IVET's efficacy in 4 individual studies. This suggests a comparable efficacy with the standardized therapy of phobic disorders [8]. The improvement in the ARET compared to the waiting list group in 2 individual studies showed statistically significant results (*P*<.05). Thus, ARET leads to a positive impact on phobic symptoms.

In addition, this study suggests the need for longer sessions in cases of exposure therapy using extended reality like VRET or ARET [16,34]. However, Javanbakht et al [32] demonstrated the ability of AR to be effective in less than 1-hour sessions. As pointed out in the results section, the mean time needed for an ARET session to give the participants the ability to walk in a room with moving large spiders with Subjective Units of Distress Scale (SUDS) below 4 was 38 (SD 12.13) minutes.

Shiban [9] discussed the added benefits of ARET over traditional exposure therapy and how the therapist can control the scenarios more easily than the IVET. Wrzesien et al [31] and Botella et al [28] supported this theoretical opinion with evidence-based inferences. Part et al [31] found that the ARET group could interact in a greater number of situations with the phobic stimulus. Unlike IVET, the ARET group could observe the phobic stimulus moving on their personal belongings, observe dead objects, put their foot near the object, and find the phobic object under different artifacts. Moreover, Botella et al [28] discussed the different variety of spiders (size, number, types, and color) and the spiders' behaviors that were easier achieved in the ARET group. Correlating the findings to the current knowledge suggests the superiority of the ARET over the IVET as patients get higher and more intense exposure due to the ability of ARET to put the patient in more scenarios in different contexts easily and without real danger. The current review of the literature suggests a better outcome in cases with more intensity and a higher number of scenarios with the feared object [35,36].

Furthermore, exposure to an increased number of scenarios can be a reason for the long-term efficacy of the treatment and less frequent recurrence [37,38]. However, it is not fully understood as Shiban et al [39] noted that an increased number of contexts would lose the positive effect after 15 days of the intervention completion. Based on our results, only Botella et al [28] had follow-up measures for the participants comparing the ARET with IVET; Botella et al [28] followed up with the patients 3 and 6 months after the completion of the treatment. Botella et al’s [28] results revealed a positive effect on the long-term efficacy of ARET, with regression in the impact of IVET.

No randomized control trials comparing ARET to VRET were identified from the search. Thus, the superiority of ARET to VRET cannot be evaluated. However, Tsai et al [16] noted a higher presence in the use of ARET compared to VRET, with statistically significant differences in the physiological effect of the use of ARET to VRET, which suggests a better outcome of ARET.

### AR and Therapeutic Alliance

As displayed in the results section, 3 studies used WAI-S as one of their outcome measures. The results showed a promising alliance between therapists and participants. These 3 compared the therapeutic alliance between ARET and IVET; the results displayed no statistically significant differences between the 2 groups in all 3 studies. However, the intervention group's results suggested a good relationship between the therapist and the participants. Compared to the traditional therapeutic approach, AR technology did not negatively or positively impact the therapeutic alliance. These results suggest ARET's good efficacy in treating small animal phobia.

Part et al [31] noted that the visual attention of the therapist using ARET was more focused on the notes and the phobic stimulus than on the patient. However, the overall attention given to the patient was nonsignificant compared to the IVET group. The nonsignificant results can be due to the small number of participants or the lack of a real effect. The current literature in this area suggests the problem in the therapeutic alliance in technology-mediated psychotherapy is due to the therapist's concerns about the alliance [20]. Part et al [31] findings propose the need for more training to use technology-mediated exposure therapy to limit attention distraction.

### AR Limitations

While AR has the potential to be a valuable tool in treating phobias, it is important to acknowledge some of its limitations to ensure the safe and effective use of AR technology. These include technical challenges, cost-effectiveness and ethical considerations.

Wrzesien et al [31] discuss some of the limitations of ARET. The interaction with the object with a stick, pen, or paper was achieved in the IVET, not the ARET group. This is one of the ARET limitations where interaction with the visual object is limited. This limitation can be overcome with mixed reality technology instead; mixed reality technology allows interaction with a visual object, but no studies were found regarding mixed reality. Moreover, Wrzesien et al [31] noted some functional issues with the system used. However, this is not a limitation of ARET but the system used in this study.

### AR Cost-Effectiveness

Baus and Bouchard [40], Bras et al [41], and Albakri et al [11] discussed the possibility of having a lower cost using AR instead of VR since fewer computer-generated objects are needed to be created; VR will need an entirely simulated environment while for AR only the stimulus object is created. However, this review failed to investigate the cost-effectiveness of AR, as no studies have been done looking into the different costs between the modalities. However, this can help us understand the need for studies looking into the cost of each modality to help us find the most cost-effective modality.

### Strengths

This study aimed to provide evidence-based inferences to a focused question looking into the efficacy of novel technology in treating phobic disorders. The use of a focused question helped in narrowing and guiding the search. In addition, it helped develop a comprehensive search strategy to cover the search area without missing related studies. Furthermore, using the PICOS approach helped standardize the selection procedure; standardization gave a low heterogenicity between the included studies.

Furthermore, all included studies are published peer-reviewed studies; using peer-reviewed studies can lower the possibility of having low-quality studies.

### Limitations

The number of studies the search reveals is small and can suggest a limitation. However, considering the comprehensive search strategy and the use of 4 massive databases, the small number revealed by the search is not due to a limitation in the search strategy. But instead, the problem is the small number of studies done in this area. The limited number of studies carried out in this area can be considered as the main limitation of this review.

Furthermore, the exclusion of grey literature in our study may be considered a limitation. It is expected that a wide range of unpublished work related to AR exists, increasing the potential of publication bias [21].

### Conclusions

Our literature review demonstrated clinically significant efficacy and a promising therapeutic alliance of ARET. However, the currently available data do not provide evidence regarding the cost-effectiveness of ARET. Further research is warranted to ascertain ARET's cost-effectiveness and examine its efficacy in other populations and anxiety conditions.

## Appendix

Multimedia Appendix 1 PRISMA checklist.

## Abbreviations

AR: augmented reality
ARET: augmented reality exposure therapy
BAT: Behavioral Avoidance Test
CASP: Critical Appraisal Skills Programme
CBT: cognitive behavioral therapy
DSM: Diagnostic and Statistical Manual of Mental Disorders
ECG: electrocardiogram
FSQ: Fear of Spiders Questionnaire
ICD: International Classification of Diseases
IVET: in-vivo exposure therapy
PICOS: Population, Intervention, Comparison and Outcomes
PRISMA: Preferred Reporting Items for Systematic Reviews and Meta-Analyses
SCR: skin conductance recordings
SPBQ: Spider Phobia Beliefs Questionnaire
SUDS: Subjective Units of Distress Scale
TAU: treatment as usual
VR: virtual reality
VRET: virtual reality exposure therapy
WAI-S: Working Alliance Inventory-Short
